# Reduce, Induce, Thrive: Bacterial Redox Sensing during Pathogenesis

**DOI:** 10.1128/JB.00128-18

**Published:** 2018-08-10

**Authors:** Michelle L. Reniere

**Affiliations:** a Department of Microbiology, University of Washington, Seattle, Washington, USA

**Keywords:** virulence, low-molecular-weight thiols, regulation, glutathione, mycothiol, bacillithiol, metabolism, iron, phagocytosis, ROS, RNS, iron regulation, pathogenesis, virulence regulation

## Abstract

The abundance of oxidants and reductants must be balanced for an organism to thrive. Bacteria have evolved methods to prevent redox imbalances and to mitigate their deleterious consequences through the expression of detoxification enzymes, antioxidants, and systems to repair or degrade damaged proteins and DNA. Regulating these processes in response to redox changes requires sophisticated surveillance strategies ranging from metal chelation to direct sensing of toxic reactive oxygen species. In the case of bacterial pathogens, stress that threatens to disrupt redox homeostasis can derive from endogenous sources (produced by the bacteria) or exogenous sources (produced by the host). This minireview summarizes the sources of redox stress encountered during infection, the mechanisms by which bacterial pathogens diminish the damaging effects of redox stress, and the clever ways some organisms have evolved to thrive in the face of redox challenges during infection.

## Abstract (precis)

The abundance of oxidants and reductants must be balanced for an organism to thrive. Bacteria have evolved methods to prevent redox imbalances and to mitigate their deleterious consequences through the expression of detoxification enzymes, antioxidants, and systems to repair or degrade damaged proteins and DNA.

## INTRODUCTION

Approximately two billion years ago, the photosynthetic activities of cyanobacteria converted the planet into an oxygen-rich environment (1). This new atmosphere brought with it significant metabolic opportunities (aerobic respiration generates more energy than fermentation) and toxic repercussions in the form of poisonous oxygenic by-products, collectively referred to as reactive oxygen species (ROS) (2, 3). The selective pressure of this environment led bacteria to develop mechanisms to mitigate oxidative stress, which results from an imbalance in metabolic homeostasis. In this review, “redox stress” refers to a general imbalance in redox homeostasis due to ROS, reactive nitrogen species (RNS), or reactive electrophilic species (RES). Prokaryotes were diverse prior to the accumulation of oxygen in the atmosphere, and therefore, the mechanisms by which bacteria cope with the challenges of oxygen toxicity are equally varied.

Redox stress is both produced by the bacteria (endogenous) and is encountered in the environment (exogenous) (2). Endogenous ROS are produced in the presence of free metals and during aerobic respiration. Free metals are toxic because they can participate in Fenton chemistry, which is the reaction of hydrogen peroxide with ferrous iron (Fe^2+^) or, less commonly, cuprous ions (Cu^1+^), to form hydroxyl radicals (4). Aerobic respiration couples the oxidation of glucose to the four-electron reduction of molecular oxygen. In the electron transport chain, electrons are passed through a series of proteins via oxidation-reduction reactions, with oxygen as the terminal electron acceptor. In this process, oxygen can be incompletely reduced, resulting in the formation of superoxide anions (O_2_^−^), hydrogen peroxide (H_2_O_2_), and hydroxyl radicals (HO·) (Table 1) (5). In addition to respiration, abundant hydrogen peroxide and superoxide are produced from the erroneous oxidation of nonrespiratory flavoproteins, such as dehydrogenases (5, 6).

**TABLE 1:** Reactive molecules

Molecule	Chemical formula	Source(s)^*a*^	Primary target(s)
Hydrogen peroxide	H_2_O_2_	Superoxide dismutase, incomplete reduction of O_2_	Fe-S clusters, proteins
Hydroxyl radical	HO·	Fenton chemistry, incomplete reduction of O_2_	DNA
Hypochlorous acid	HOCl	MPO	Proteins
Nitric oxide	˙NO	iNOS	Proteins, metals
Nitrogen dioxide	˙NO_2_	Spontaneous reaction of ˙NO and O_2_^−^	Proteins
Nitroxyl radical	NO^−^	Product of NorV-mediated NO reduction	Proteins
Peroxynitrite	ONOO^−^	Spontaneous reaction of ˙NO and O_2_^−^	Proteins, DNA, metals
Superoxide anion	O_2_^−^	NOX, incomplete reduction of O_2_	Fe-S clusters

a MPO, myeloperoxidase; iNOS, nitric oxide synthase; NOX, NADPH oxidase.

Endogenous sources of redox stress are evolutionarily ancient and important to consider because they were the original driving force behind the evolution of redox homeostasis pathways. For example, the model Gram-negative organism Escherichia coli produces approximately 10 μM/s hydrogen peroxide and 5 μM/s superoxide during aerobic respiration (6, 7). This endogenous production of ROS can cause significant redox stress if uncontrolled, as evidenced by the substantial aerobic growth defects exhibited by mutant strains unable to detoxify ROS (8, 9). Further, E. coli mutants that cannot detoxify ROS are only able to grow because they induce a stress response that chelates iron and repairs DNA damage, as discussed below (10). While bacteria evolved detoxification strategies to survive in an aerobic environment, these ancient pathways were then repurposed to thrive in the host during infection. This minireview focuses on exogenous sources of redox stress encountered by bacterial pathogens, the mechanisms by which they manage this stress, and examples of bacteria that exploit host defenses to coordinate their metabolic adaptation and activate virulence programs.

## EXOGENOUS SOURCES OF REDOX STRESS

**FIG 1:**
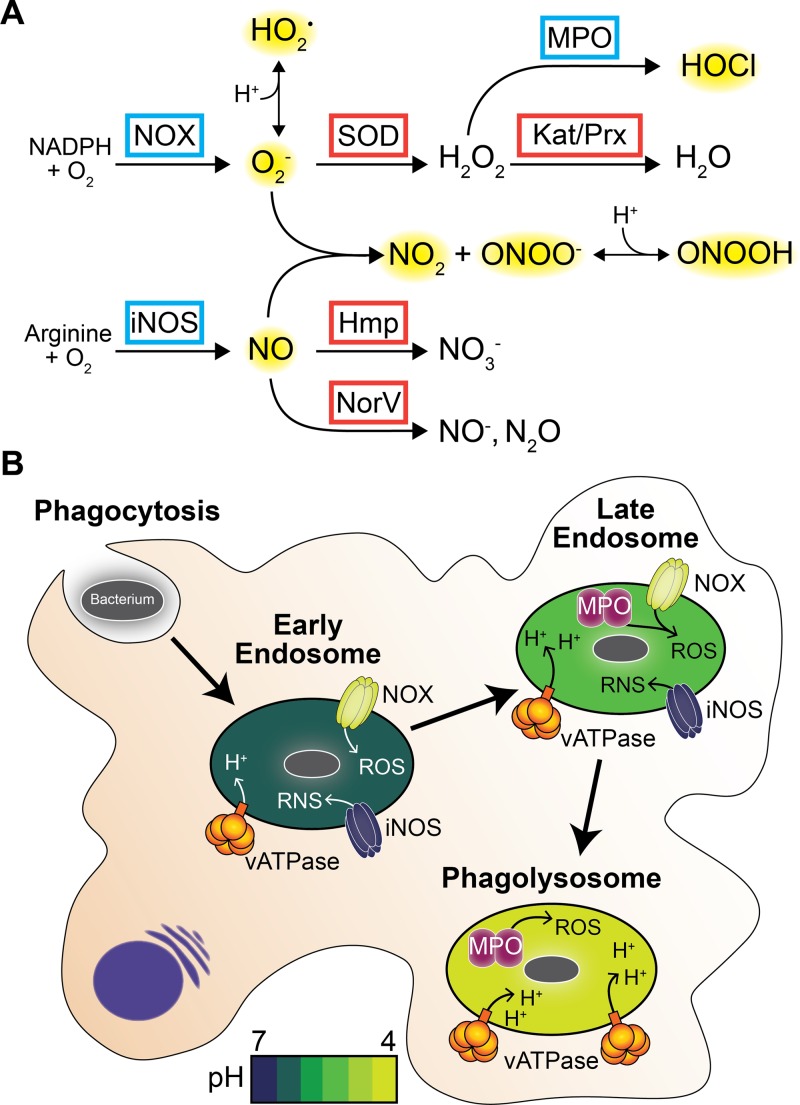
Host defense mechanisms against intracellular bacterial pathogens. (A) Host-derived antimicrobial ROS and RNS (highlighted in yellow). Host proteins are in blue boxes, and bacterial detoxification enzymes are in red boxes. NADPH oxidase (NOX) and inducible nitric oxide synthase (iNOS) are recruited to the phagosome in the respiratory burst. Myeloperoxide (MPO) is a significant component of neutrophil granules but is also found in phagolysosomes (97). Superoxide dismutase (SOD), catalase (Kat), and peroxiredoxins (Prx) detoxify ROS, while flavohemoglobin (Hmp) and flavorubredoxin (NorV) detoxify RNS. At low pH, peroxynitrite will be protonated (ONOOH [24]). Similarly, superoxide is protonated in the phagolysosome to form the reactive HO_2_˙ species (13). (B) Simplified schematic depicting macrophage phagosomal maturation (97). The pH of the phagosome steadily decreases, according to the pH scale shown, via recruitment of the vacuolar ATPase (vATPase). The bacterium is in gray. Not drawn to scale.

Exogenous sources of redox stress are abundant, particularly for bacterial pathogens that encounter assaults from the mammalian immune system during infection. Phagocytes, primarily macrophages and neutrophils, are recruited to sites of infection to ingest invading bacteria and bombard them with oxidants in a process referred to as the “respiratory burst.” The host NADPH oxidase (NOX2) complex is activated by phagocytosis of bacteria (11), generating superoxide radicals and hydrogen peroxide in the phagosome (Fig. 1). Nitric oxide synthase (iNOS) is also induced, producing nitric oxide (˙NO) that reacts with superoxide in the phagosome to form peroxynitrite (ONOO^−^), nitrogen dioxide (˙NO_2_), and other toxic RNS. Myeloperoxidase in neutrophils then consumes the hydrogen peroxide produced by NOX2 to generate hypochlorous acid (HOCl), a strong two-electron oxidant (12). ROS generated by NOX2 are also important components of neutrophil extracellular traps that ensnare and kill extracellular bacterial pathogens (13).

Chronic granulomatous disease (CGD) is a genetic disorder in which individuals lack functional NOX2 and therefore do not mount an effective respiratory burst. The importance of respiratory burst oxidants in microbial killing is underscored by the multitude of recurrent bacterial infections suffered by patients with CGD and their significantly decreased life expectancy (14, 15). CGD patients are particularly susceptible to infection by Staphylococcus aureus, Serratia marcescens, Burkholderia cepacia, and fungal pathogens, with S. aureus being the most frequently isolated bacterium (16). CGD patients are also prone to infection by opportunistic pathogens that rarely cause disease in immunocompetent individuals, highlighting the importance of the respiratory burst to human health.

Bacteria have evolved various mechanisms to cope with the respiratory burst; some actively manipulate the phagosome to prevent accumulation of ROS and RNS, while others escape this compartment entirely and replicate in the host cytosol. However, pathogens are confronted with other sources of redox stress in the mammalian cytosol, such as mitochondrially derived ROS, RNS, and methylglyoxal, an RES that causes alkylation of protein thiols and induces the production of ROS and RNS (17, 18). In addition, mitochondrially derived hydrogen peroxide can oxidize the cytosolic iron storage protein ferritin, releasing free iron that can produce ROS via Fenton chemistry (19).

ROS and RNS are deleterious to nearly all biomolecules, and their effects manifest in distinct fashions (Table 1). The superoxide anion is a charged molecule at physiological pH, so it cannot diffuse freely across membranes; therefore, its effects are restricted to the local microenvironment in which it was formed (8). Superoxide primarily causes damage by oxidizing iron-sulfur clusters, which results in the release of iron. Although catalytically versatile, iron-sulfur clusters are a dangerous redox-sensitive cofactor; oxidation not only damages the cofactor and inhibits enzymatic activity, but it also releases free iron that can propagate the oxidative stress (20). Hydrogen peroxide can oxidize iron-sulfur clusters, as well as lipids and protein cysteine residues, and is capable of generating protein carbonylation (8, 21). The hydroxyl radical is the most potent ROS and can damage the majority of biomolecules, although the most significant impact of this radical is likely in creating DNA lesions (8). Hypochlorous acid oxidizes proteins, primarily on cysteine and methionine residues, leading to protein unfolding and aggregation (Table 1) (12). Nitric oxide can also modify cysteine residues (*S*-nitrosylation) and react with transition metals (22), while peroxynitrite is a strong oxidizing and nitrating compound that oxidizes protein thiols, purine nucleotides of DNA, and the transition metal centers of metalloproteins (23, 24). The actual consequences of all these ROS and RNS are dependent on several variables, including the density of the bacterial culture, its growth phase and metabolic activity, and the concentration of ROS (25). For example, at low concentrations, hydrogen peroxide primarily targets DNA and has a bacteriostatic effect, whereas at high concentrations, peroxide is bactericidal due to the broad oxidation of protein thiols and iron-sulfur clusters.

## REDUCE: MECHANISMS TO REDUCE REDOX STRESS BEFORE DAMAGE OCCURS

To combat both endogenous and exogenous oxidative assaults, pathogenic bacteria have constitutive detoxification methods and inducible systems. Mechanisms to limit redox stress include sequestration of heavy metals, repair of damaged DNA and proteins, production of low-molecular-weight thiol antioxidants, and detoxification enzymes that consume ROS.

To prevent aberrant endogenous ROS accumulation, unincorporated iron is kept at very low concentrations by regulating its uptake, efflux, and storage. Iron can be stored in three types of proteins: ferritin, bacterioferritins, and Dps proteins (26). In addition to sequestering iron, Dps proteins also bind DNA to physically protect it from oxidative damage (27). If DNA is damaged, these oxidative lesions must be repaired. This was demonstrated with the Gram-negative pathogen Salmonella, in which the DNA repair enzyme RecA is required for resistance to hydrogen peroxide *in vitro* and during infection, indicating that host-derived ROS are sufficient to damage bacterial DNA (25, 28).

Cysteine is an essential amino acid but is prone to metal-catalyzed auto-oxidation yielding cystine and toxic ROS (29). Protein thiols can therefore function as redox-sensitive switches that can be reversibly oxidized to sulfenic acid (-SOH) or disulfides, thereby altering the activity of a protein in response to oxidation (30). In contrast, protein carbonylation and cysteine overoxidation to sulfinic acid (-SO_2_H) or sulfonic acid (-SO_3_H) are irreversible and require degradation machinery to remove or repair the damaged proteins. Thus, stress and shock proteins are induced during redox stress and serve as protein chaperones or function in reconstituting overoxidized proteins (31). One well-characterized example is the bacterial heat shock protein 33 (Hsp33), which is an oxidation-sensitive chaperone that is activated upon redox stress to sequester unfolding proteins and prevent the accumulation of protein aggregates (32).

Although less reactive than cysteine, methionine residues can also be oxidized in the presence of ROS, converting them to methionine sulfoxides that alter protein structure and inactivate or modulate protein function. The repair of this oxidation requires dedicated methionine sulfoxide reductase (Msr) enzymes (33). In the gastric pathogen Helicobacter pylori, methionine oxidation of catalase (KatA) destabilizes its secondary structure and destroys its activity (34). Therefore, Msr and the protein chaperone GroEL are required to repair oxidatively damaged KatA and restore its activity in order for H. pylori to survive the neutrophil respiratory burst (33, 34). Staphylococcus aureus strains deficient in Msr are also more susceptible to neutrophil killing (35). Together, these data suggest that methionine oxidation occurs in the phagosome of neutrophils and must be repaired for the ingested bacteria to survive.

Low-molecular-weight (LMW) thiols are small molecules containing a reactive sulfhydryl that participates in thiol-disulfide exchange reactions to detoxify ROS and maintain the cytoplasm in a reduced state. During oxidative stress, LMW thiols form reversible mixed disulfides with reactive protein thiols to protect them from overoxidation or to alter their activity, a process referred to as *S*-glutathionylation (36). Many LMW thiols are also storage forms of cysteine that are more resistant to autoxidation (29, 37). The most abundant LMW thiols produced by prokaryotes are glutathione (GSH), mycothiol (MSH), bacillithiol (BSH), cysteine, and coenzyme A (29). GSH is the primordial LMW thiol and is produced by all eukaryotes with mitochondria, most Gram-negative bacteria, and some Gram-positive bacteria (38). GSH is a tripeptide consisting of glycine, glutamine, and cysteine linked via a unique gamma-glutamyl bond that makes it resistant to canonical cellular peptidases (39). MSH is a cysteine glycoconjugate and is the major LMW thiol produced by actinobacteria, including the important human pathogen Mycobacterium tuberculosis (37). M. tuberculosis mutants with altered MSH-to-MSSM (reduced mycothiol-to-oxidized mycothiol) ratios are impaired during infection, highlighting the importance of thiol homeostasis to pathogenesis (40). BSH is a glycoside formed between l-cysteinyl-d-glucosamine and malic acid that is produced by Bacillus species and several staphylococci and streptococci (41–43). GSH and its analogs in bacteria have been reviewed by a leader in the field, and the reader is directed to that paper for more details on the structures and functions of these LMW thiols (29).

In addition to LMW thiols, bacteria produce detoxification enzymes that consume ROS. Superoxide dismutase (SOD) is a metalloenzyme that converts superoxide to hydrogen peroxide and can be localized in the bacterial cytoplasm to scavenge endogenous superoxide or extracytoplasmic to detoxify exogenous superoxide (44). Hydrogen peroxide is then reduced to water by catalase, peroxiredoxin, or glutathione peroxidases (45). Thioredoxins and glutaredoxins are small oxidoreductases that perform thiol-disulfide exchange reactions, keeping protein thiols in the cell reduced. These are then recycled by thioredoxin reductases and GSH, respectively (46).

The most well-characterized RNS detoxifying enzymes are flavohemoglobin (Hmp) and flavorubredoxin (NorV). Hmp is a heme-binding nitric oxide dioxygenase that detoxifies nitric oxide to nitrate (NO_3_^−^) via a reductase domain that supplies electrons to the active site (47). The enteric pathogen Salmonella enterica serovar Typhimurium and uropathogenic E. coli (UPEC) strains that lack Hmp are attenuated for virulence (48). NorV reduces nitric oxide to nitroxyl (NO^−^), which rapidly decomposes to nitrous oxide (N_2_O, Fig. 1A). Although NorV is an oxygen-sensitive enzyme utilized during anaerobic growth, it is capable of reducing NO in intracellular enterohemorrhagic E. coli (EHEC) and is required for EHEC survival in macrophages (49). Together, these data suggest that detoxifying NO is critical for infection of Salmonella, UPEC, and EHEC bacteria.

## INDUCE: INDUCIBLE DEFENSE REGULONS

Basal expression of antioxidants and scavenging systems is required for surviving constitutive endogenous redox stressors, while the ability to induce detoxification systems is important for combatting exogenous sources of redox stress as they arise. Several families of transcription factors directly sense alterations in the redox environment and adjust the bacterial response appropriately (Table 2). Three transcription factors that directly sense peroxides have been particularly well characterized, OxyR, PerR, and OhrR (45).

**TABLE 2:** Redox-responsive regulators

Regulator	Stress stimulus	Sensory mechanism	Reference(s)
NorR	Nitric oxide	Nitrosylation of coordinated iron	60
NsrR	Nitric oxide	Nitrosylation of iron-sulfur cluster	61
OhrR	Organic peroxide	Cysteine oxidation	98
OxyR	Hydrogen peroxide	Cysteine oxidation	99, 100
PerR	Hydrogen peroxide	Histidine oxidation	55
Rex	NADH/NAD^+^ ratio	NAD^+^ enhances DNA binding	65
SoxR	Redox-cycling compounds	Oxidation of iron-sulfur cluster	67
SpxA	Disulfide stress	Cysteine oxidation	63

The first peroxide-sensing transcription factor described was OxyR in Salmonella, which has since been found to be widely conserved in Gram-negative and some Gram-positive bacteria (50, 51). Experiments in E. coli demonstrated that OxyR is activated by 1 μM extracellular hydrogen peroxide, which equates to intracellular peroxide levels of 200 nM (5). Depending on its oxidation state, OxyR regulates approximately 40 genes in S. enterica (52). In the Gram-negative pathogen Francisella tularensis, OxyR is required to activate the genes encoding catalase and SOD in response to oxidative stress during infection (Table 3). F. tularensis mutants lacking *oxyR* therefore exhibit a growth defect in macrophages and are attenuated in virulence (53).

**TABLE 3:** Examples of redox regulators involved in virulence

Pathogen	Regulator (family)	Function *in vivo*	Reference(s)
Vibrio cholerae	NorR	NorR is required to upregulate *hmp* to detoxify NO during infection	101
Salmonella enterica serovar Typhimurium	NsrR	NsrR-regulated genes are required for nitrosative stress resistance and infection	102
Staphylococcus aureus	MgrA (OhrR)	Δ*mgrA* mutant strains are less virulent due to increased expression of surface proteins and decreased capsule expression	103, 104
	Rex	Rex derepression is required for upregulation of lactate dehydrogenase and NO resistance during infection	65, 105
Mycobacterium tuberculosis	OxyS (OxyR)	Oxidation of OxyS derepresses catalase (*katG*) expression; *katG* is required for infection, but point mutations result in isoniazid resistance	106, 107
	MosR (OhrR)	MosR derepresses an oxidoreductase during macrophage infection	59
Francisella tularensis	OxyR	Δ*oxyR* mutant is deficient in intracellular survival and virulence due to enhanced susceptibility to oxidative stress	53
Streptococcus pyogenes	PerR	PerR-dependent gene expression is required to survive the oxidative burst in macrophages	108
	SpxA	Δ*spxA1* mutant is attenuated, Δ*spxA2* mutant is hypervirulent	64
Listeria monocytogenes	PrfA	GSH allosterically activates master virulence regulator	75
	SpxA	Δ*spxA1* mutant is attenuated, Δ*spxA2* mutant is fully virulent	109
Pseudomonas aeruginosa	SoxR	SoxR is required for virulence	110
Corynebacterium diphtheriae	DtxR	DtxR derepresses diphtheria toxin under iron-limiting conditions, damaging host	79

PerR, named for its peroxide sensing, is a DNA-binding protein in the Fur family of metal-responsive transcription factors in which repression is relieved upon oxidation of the metal-coordinating histidine residues (Table 2) (54). PerR senses peroxides via metal-catalyzed oxidation of the two histidine residues required to bind iron (55). OhrR (organic hydroperoxide resistance regulator) is a transcriptional repressor whose repression is relieved by oxidation of a conserved cysteine residue in the presence of organic hydroperoxide stress or hypochlorite stress (56, 57). The OhrR subfamily of proteins is extensive and includes regulators, such as MgrA, SarZ, SarA, MosR, RosR, and QsrR that control gene expression in response to the oxidation of one or more protein thiols (58).

Although the particulars vary among different bacteria, in general, OxyR, PerR, and OhrR family proteins regulate genes required to adapt to redox stress. Some of these include genes encoding catalase, thioredoxins, heme biosynthesis machinery, glutathione reductases, Fur, ferritin, and bacterioferritin. Additionally, the OhrR/SarA/MgrA subfamily regulates genes involved in virulence and antibiotic resistance (Table 3). For more comprehensive analyses of the molecular details of redox sensing by OxyR, PerR, and the OhrR family, readers are directed to several excellent recent reviews (45, 58, 59).

Nitric oxide and its analogs are directly sensed by NorR and NsrR (Table 2). In enterobacteria, NsrR and NorR regulate enzymes required to detoxify nitrosative stress, including those encoded by *hmp* and *norV* (48). NsrR is a transcriptional repressor that is inactivated by nitrosative stress, whereas NorR is a transcriptional activator that is activated by nitric oxide (60, 61). Both proteins are required to detoxify nitrosative stress *in vivo* (Table 3).

In addition to the proteins that directly sense peroxides and nitric oxide, redox homeostasis is coordinated by proteins that sense disulfide stress, NADH, and redox-cycling compounds (Table 2). SpxA is a disulfide stress regulator of the arsenate reductase (ArsC) family of proteins that is regulated by redox changes via an N-terminal C-X-X-C redox switch. Oxidized SpxA positively regulates approximately 275 genes in the model Gram-positive bacterium Bacillus subtilis, including genes encoding bacillithiol biosynthesis, thioredoxin, and components of proteolytic pathways (62, 63). Many Firmicutes encode more than one SpxA family protein that can function cooperatively or independently. For example, in Streptococcus pyogenes, the two SpxA family proteins function antagonistically during infection, such that an *spxA1*-deficient strain is attenuated, while the deletion of *spxA2* results in a hypervirulent strain (Table 3) (64).

Rex is a transcriptional repressor that responds to NADH/NAD^+^ ratios to regulate metabolic pathways that regenerate NAD^+^ in Gram-positive bacteria (65). SoxR is a MerR family sensor protein containing two iron-sulfur [2Fe-2S]^2+^ clusters, the oxidation of which alters the conformation of the protein and its interaction with promoter DNA (52). The SoxR protein was named such due to its importance in the superoxide response in E. coli strains challenged with 1,1′-dimethyl-4,4′-bipyridinium dichloride (Paraquat) (66). However, this response is indirect, and SoxR actually responds to redox cycling compounds (such as Paraquat) via direct oxidation of the bound iron-sulfur cluster (67).

Iron concentrations are critical to control in order to mitigate redox damage, so while not exactly redox-responsive regulators, the metalloregulatory proteins Fur (ferric uptake regulator) and DtxR (diphtheria toxin repressor) are critical to overall redox homeostasis. Fur and DtxR are iron-sensing transcriptional repressors that control the expression of genes encoding iron acquisition systems, iron-dependent enzymes, metabolic proteins, and virulence factors (68). In fact, the abundance of free iron in the cytoplasm of M. tuberculosis is a major determinant of redox stress during infection (40).

Metal availability is so critical to infection outcomes that an arms race has developed between the host and the pathogen surrounding the regulation of metal-dependent enzymes. One example of this comes from the Lyme disease agent Borrelia burgdorferi, which utilizes manganese in place of iron as a protein cofactor in order to bypass the requirement for iron altogether (69). However, neutrophils attack invading pathogens with a one-two punch, including both ROS generated during the oxidative burst, as well as delivery of the manganese-binding protein calprotectin (70). Calprotectin is the most abundant protein in the neutrophil cytoplasm and functions to chelate manganese and zinc in an effort to starve invading bacteria (71). In the case of the Gram-positive extracellular pathogen S. aureus, calprotectin can limit bacterial growth via inhibition of a manganese-dependent SOD and a simultaneous increase in superoxide concentrations inside the bacterium (72). However, S. aureus counters this attack by producing two SODs, one of which is upregulated in response to calprotectin and can function with either manganese or iron, allowing the bacterium to adapt to the host immune response (73). In addition, S. aureus alters its carbon utilization to reduce manganese demand and resist calprotectin-mediated manganese starvation (74).

## THRIVE: BACTERIAL PATHOGENS USE HOST CUES TO MODULATE VIRULENCE

Examples are now emerging of bacteria that have coopted host defenses for their own signaling pathways, metabolic adaptation, and virulence (75–77). In a process termed “nutritional immunity,” the host sequesters iron within proteins to withhold it from invading pathogens (78). Bacterial pathogens have evolved to sense the absence of free iron to detect their entry into a vertebrate host (70). For example, in E. coli, the type of SOD produced depends on the microenvironment, whereby Fe-SOD is produced constitutively, and the oxidation-resistant Mn-SOD is induced when the bacterium is exposed to redox stress (2). As discussed previously, Fur and DtxR are iron-sensing transcription factors that regulate iron homeostasis and virulence and are able to identify this low-iron environment in order to adjust the transcriptional response. S. aureus secretion of alpha-toxin and leukotoxin is regulated by Fur to promote pathogenesis in the iron-limiting host. Similarly, expression of the diphtheria toxin in Corynebacterium diphtheriae is regulated by DtxR in an iron-dependent manner (Table 3) (79).

Some intracellular pathogens have evolved detection methods for the ubiquitous and highly abundant cytosolic antioxidant GSH. When phagocytes generate ROS and RNS to destroy invading pathogens during the respiratory burst, they simultaneously produce and import GSH as a self-protection mechanism, and up to 10 mM GSH can accumulate in the cytosol (39, 80, 81). Burkholderia pseudomallei is a Gram-negative facultative intracellular pathogen that causes melioidosis, and its virulence is completely dependent on the expression of a type VI secretion system (T6SS). During infection, B. pseudomallei senses host-derived GSH via binding to a histidine kinase sensor VirA, which then activates the expression of the T6SS (76). Listeria monocytogenes also senses host GSH but by a distinct mechanism. L. monocytogenes is a Gram-positive intracellular pathogen and the causative agent of the serious foodborne illness listeriosis. Pathogenesis of L. monocytogenes requires activation of the master virulence transcriptional regulator PrfA. It was recently demonstrated that both host-derived and bacterially derived GSH allosterically bind PrfA to transcriptionally activate virulence genes in L. monocytogenes (75, 82–84). Moreover, bacterial production of GSH increases specifically in the host cell. These studies further suggest that PrfA itself acts as a redox sensor in which activation requires reduction of the protein thiols as well as abundant GSH (Table 3). It is clear from these examples that intracellular pathogens capitalize on the host cytosol being a GSH-rich niche and have hijacked this LMW thiol as a cue that they are in the host cytosol in order to trigger appropriately timed virulence gene expression.

In addition to utilizing host LMW thiols to activate virulence gene expression, host-derived GSH is also used by pathogens to inhibit specific virulence factors. L. monocytogenes secretes the pore-forming toxin listeriolysin O (LLO) to mediate escape from the phagocytic vacuole, but the activity of LLO in the cytosol has the potential to lyse the host cell, destroying the replicative niche of the bacteria. One pathway to prevent LLO activity in the cytosol is the *S*-glutathionylation of LLO by host-derived GSH, which inhibits its activity and physically blocks its association with membranes (85). Another example comes from Yersinia pestis, the causative agent of plague, which uses a type III secretion system (T3SS) to transport effector proteins into host cells. The T3SS cap protein LcrV is *S*-glutathionylated in macrophages, thereby blunting host cell death and enhancing plague pathogenesis (86). Together, these examples illustrate how diverse bacterial pathogens have evolved distinct mechanisms to utilize host-derived GSH to regulate virulence factor production and activity.

## BACTERIAL PATHOGENS EXPLOIT HOST DEFENSES FOR THEIR BENEFIT

Bacterial pathogens exploit the host response to coordinate metabolic changes that promote adaptation to the host environment. The simplest example of this is Bacillus anthracis, the causative agent of anthrax, which persists in the environment as dormant endospores. B. anthracis endospores are highly resistant to oxidative stress and germinate in response to superoxide exposure in phagocytes, a critical first step of infection (13, 87). Neisseria gonorrhoeae, the causative agent of gonorrhea, is a Gram-negative extracellular pathogen that forms biofilms, allowing the bacterium to persist within a host and cause chronic infections. Gonococci in the substratum of the biofilm near the surface of host cells can sense the levels of nitric oxide produced by the endothelial or epithelial cells. In response, the bacteria activate genes required for anaerobic respiration and reduce nitric oxide concentrations (88). Commensal E. coli strains also capitalize on the host inflammatory response to gain a growth advantage in the gastrointestinal tract. Nitrate generated by the host as a by-product of iNOS activity feeds E. coli anaerobic respiration in the gut, allowing them to outcompete other commensal bacteria (89).

Another example of bacterial metabolic remodeling during infection comes from *S*. Typhimurium, an enteric pathogen that causes massive acute inflammation and diarrhea. ROS generated by the influx of phagocytes to the site of inflammation convert thiosulfate to tetrathionate, which can then be used by *S*. Typhimurium as a terminal electron acceptor, allowing it to outcompete commensal bacteria (90). *S*. Typhimurium also expresses multiple manganese transporters and manganese-dependent SOD and catalase enzymes to survive in the presence of abundant calprotectin that is secreted in the inflamed gut (91).

H. pylori is a successful gastric pathogen that exploits host defenses to establish chronic infections that can persist for decades. Upon H. pylori invasion of the gastric epithelium, the host defenses are activated, including upregulated ROS production and delivery of calprotectin to the site of infection by innate immune cells. In response, H. pylori first senses the host-derived ROS to drive chemotaxis and promote the colonization of new glands, enabling chronic colonization (92, 93). Second, zinc sequestration by calprotectin inhibits the inflammation-promoting type IV secretion system, resulting in reduced inflammation and, ultimately, increased bacterial persistence (94). Additionally, calprotectin-dependent manganese sequestration can result in lipid A modification, which leads to enhanced biofilm formation and increased bacterial fitness (95).

These examples demonstrate that bacterial pathogens have evolved to not only survive the redox stressors encountered during infection, but in some cases, to utilize them as host-specific signals. This can be described as a “hormetic” response in which a low dose of a poison actually has beneficial effects by stimulating an advantageous response (96). For example, researchers identified OxyR based on the fact that Salmonella spp. preexposed to 60 μM hydrogen peroxide were subsequently resistant to 10 mM peroxide (51). However, in this case, the host-mediated redox changes are detected by the bacteria and “prime” the system to induce not only the bacterial redox defense mechanisms but also virulence genes and metabolic adaptation that promote pathogenesis. To understand this complex interaction, future research will explore the host microenvironments experienced by bacterial pathogens and their adaptive responses.
